# Application effects analysis of a nurse-led and family-involved pain management program for children with limb fractures

**DOI:** 10.3389/fped.2026.1908282

**Published:** 2026-10-28

**Authors:** Yanqing Xu, Haiyan Lin, Yuting Zhu, Jinrun Lin

**Affiliations:** Department of Orthopedics, Fujian Children’s Hospital (Fujian Branch of Shanghai Children’s Medical Center), College of Clinical Medicine for Obstetrics & Gynecology and Pediatrics, Fujian Medical University, Fuzhou, Fujian Province, China

**Keywords:** children, family-involved, fractures, nurse, pain

## Abstract

**Purpose:**

This study aimed to assess the effectiveness of a nurse-led, family-involved pain management program in children with limb fractures.

**Methods:**

A total of 120 children undergoing limb fracture surgery at Fujian Children's Hospital from January 1, 2024 to May 31, 2025 were enrolled. A controlled before-after quasi-experimental design was adopted, and the children were divided into groups according to the order of admission: the control group received routine nursing care, whereas the intervention group received a nurse-led, family-involved pain management program for children with limb fractures. The primary outcomes, pain scores at 6 and 24 h postoperatively, were compared between the two groups. Secondary outcomes included length of hospital stay, complications, fear of movement, family anxiety, and functional exercises compliance.

**Results:**

A total of 120 children were enrolled (60 in the control group and 60 in the intervention group). Primary outcome showed postoperative pain scores at 6 h and 24 h were both significantly lower in the intervention group (6h: U = 1,073.5, *P* < 0.001, r = 0.367, median difference = 0.99994, 95% CI:0.00005–1.00000; 24h: U = 402, *P* < 0.001, r = 0.685, median difference = 2.00, 95% CI: 2.00–2.00). Secondary outcomes showed shorter hospital stays [3 (IQR: 2, 3) vs. 4 (IQR: 2, 5); U = 1,224, *P* = 0.002], lower TSK scores (24.65 ± 2.43 vs. 37.10 ± 2.71; MD = 12.45, 95% CI: 11.520–13.380, *P* < 0.001), and a higher compliance rate with functional exercises (86.7% vs. 58.3%; *χ*^2^ = 12.079, *P* = 0.001). Preoperative SAS scores were comparable between groups, while postoperative SAS scores were significantly lower in the intervention group [42 (IQR: 40, 44) vs. 63.5 (IQR: 63, 63.75); U = 12, *P* < 0.001]. No complications were reported.

**Conclusion:**

The nurse-led, family-involved pain management program for children with limb fractures was associated with meaningful improvements in postoperative pain, reduced fear of movement, better exercise compliance, and decreased family anxiety compared with routine nursing care. These findings suggest that the program may be a promising approach for postoperative management in children with limb fractures.

## 1 Introduction

Pediatric fractures are the most common accidental injuries in children, with falls being the leading cause. The annual incidence is approximately 120–361 per 10,000 children, and up to one-fifth of children have experienced at least one fracture before adulthood (1–3). Limb fractures account for 80% of all pediatric fractures, with the majority involving the upper extremity and metaphyseal fractures being the most prevalent type (4, 5).

Pain is a common and prominent symptom in children with fractures. Preoperative trauma, surgery, postoperative fixation, and functional exercises can all induce varying degrees of pain and discomfort (6, 7). Effective pain relief can improve mobility, promote rehabilitation, and accelerate the return to normal daily activities (8). However, approximately 20%–80% of children continue to experience moderate to severe pain postoperatively, and optimal postoperative pain management remains a global challenge (9).

Nurses’ responsibilities span the entire treatment process, from preoperative preparation to discharge follow-up. As the frontline of clinical practice, nurses have the most frequent and sustained contact with children and their families throughout the perioperative period (10). Moreover, nurses are directly involved in pain management, wound care, and patient education, making them key contributors to recovery (11). Given the young age of pediatric patients, their family members provide emotional and practical support and assist with functional exercise adherence, making them key partners in the treatment and rehabilitation process (11, 12).

Given the importance of nurses and family involvement, along with the current lack of reported studies in this area, our hospital has established a nurse-led and family-involved pain management program for pediatric patients with limb fractures. This quasi-experimental study aimed to evaluate the effectiveness of this program in this patient population.

## 2 Materials and methods

### 2.1 Study subjects​

Study subjects were selected from the clinical data of 120 pediatric patients with limb fractures who underwent surgical treatment and were hospitalized in the Department of Orthopedics, Fujian Provincial Children's Hospital, from January 1, 2024 to May 31, 2025. All eligible patients during each study period were enrolled consecutively. Using a controlled before-after quasi-experimental study method, the patients were divided into an intervention group and a control group according to the order of admission. The control group received routine nursing care, while the intervention group was intervened with the nurse-led and family-involved pain management program for pediatric patients with limb fractures. Informed consent from the pediatric patients and their family members was obtained for all participants in accordance with the Declaration of Helsinki. This study had obtained approval from the Ethics Committee of Fujian Children's Hospital (Ethics No.2025ETKLRK04014).

### 2.2 Inclusion and exclusion criteria​

Inclusion Criteria: Voluntarily participate in and actively cooperate with nursing interventions, and fully understand this study; possess normal communication skills and basic expression abilities; pediatric patients with limb fractures who underwent surgical treatment; children aged over 6 years old.

Exclusion Criteria: Incomplete clinical data or multiple fractures; pediatric patients who refused surgical treatment or received conservative treatment; cognitive impairment or mental abnormalities.

### 2.3 Intervention methods

The intraoperative anesthesia and analgesia regimen, antibiotic use, and wound care procedures were identical between the two groups.

#### 2.3.1 Control group

The control group received a routine nursing care, which included the following components:

On the day of admission, the primary nurse provided standardized admission education, covering the ward environment, introduction of the attending physician and primary nurse, and hospital rules and regulations. One day before surgery, the primary nurse provided the family with a printed preoperative education sheet and verbally explained the fasting and fluid restriction times, key points for surgical cooperation, and precautions, instructing family members on how to assist their children in completing preoperative preparations.

After return to the ward, vital signs (heart rate, respiratory rate, blood pressure, and oxygen saturation) were monitored according to the ward's standard protocol—every hour for the first 6 h post-surgery, and then every 8 h thereafter. Pain was assessed by the primary nurse using the Numerical Rating Scale (NRS) at three time points: immediately upon return to the ward, at 6 h post-surgery, and at 24 h post-surgery, or whenever the child showed signs of significant distress. When the NRS score was ≥ 3, the nurse administered ibuprofen suppositories according to the physician's order, with a reassessment 30 min after administration. If the pain score remained below 3 for two consecutive assessments, the frequency was reduced to once every 24 h. When the NRS score was ≥ 3, a local ice compress was applied with the assistance of the nurse for 30 min each time.

After full recovery from anesthesia, a trial of water was initiated at 4 h post-surgery. If no nausea or vomiting occurred, the child was transitioned to a liquid diet, with gradual progression to a regular diet after 6 h. Functional exercise guidance: On the morning of the first postoperative day, the primary nurse provided one session of verbal instruction accompanied by illustrated materials (approximately 3–5 min), explaining how family members could assist the child with functional exercises. The actual execution was left to the family's discretion, without a standardized protocol. Discharge instruction: On the day of discharge, the primary nurse provided routine discharge education, covering wound care, follow-up schedule, and contact information.

#### 2.3.2 Intervention group

On the basis of the control group, a nurse-Led and family-Involved pain management program was adopted. The differences in nursing protocols between the two groups are presented (Table 1). The specific implementation plan is as follows:

**Table 1:** Comparison of the care protocols between the control and intervention group.

Item		Control Group	Intervention Group
Admission education		Identical	Identical
Preoperative preparation	preoperative fasting	Identical	Identical
	preoperative education	a printed preoperative education sheet	A printed preoperative education sheets + 15-min psychological counseling (family and child) + 15-min family training
Postoperative vital signs monitoring		Identical	Identical
Postoperative pain assessment	pain assessment	Assessed at return to ward, 6 h and 24 h post-surgery; additional assessments if the child shows signs of significant distress	Assessed at return to ward, 6 h and 24 h post-surgery; additional assessments if the child shows signs of significant distress + Family members assessed
	pain treatment	Ibuprofen administered when pain score ≥3，if no relief after 30 min, nurse applies ice compress + family members use music, games, and other distraction techniques	Ibuprofen administered routinely upon return to ward + If pain score <3: instruct family to apply ice compress 2–3 times daily and use music, games, and other distraction techniques + Ibuprofen administered when pain score ≥3
Dietary guidance		Identical	Identical
Functional exercise guidance		Verbal instruction + picture display	Verbal instruction + picture display + nurse demonstration +"Little Warrior"game (rewards for task completion)
Discharge instruction		Identical	Identical

A pain management team was established, consisting of head nurses, pain specialist nurses, orthopedic specialist nurses, and physicians. The team was led by pain specialist nurses with collaboration from other disciplines. Nurses guided family members to record the children's manifestations to assist in pain assessment, and jointly helped alleviate the children's pain through individualized treatment measures. Pain specialist nurses formulated standardized management protocols, and continuous improvement was promoted through the Plan-Do-Check-Act (PDCA) cycle.

Ibuprofen suppositories shall be routinely administered when the pediatric patients return to the ward after surgery. Medical staff shall adopt individualized treatment measures according to the pediatric patients’ age, cognitive level, and other pain—influencing factors. Simple training will be provided to the family members of pediatric patients, including: potential preoperative and postoperative conditions, basic pain assessment methods, and feasible intervention measures. More specifically: When the pain score is <3, family members can achieve analgesic effects through distraction techniques based on the child's preferences, such as selecting the child's favorite music, video games, or audio books; local ice compress can alleviate the pain experience. When the pain score is ≥3, family members should immediately notify the attending physician and use analgesics (e.g., ibuprofen) as prescribed by the doctor. Regular pain score follow—ups should be conducted after medication administration to monitor the analgesic effect. Nursing staff enhance the nurse-patient relationship through hand-drawn illustrations, storytelling, and other age-appropriate methods. They help pediatric patients distract their attention, and alleviate negative emotions by organizing games—with game selection tailored to the child's age and cognitive level. Additionally, nursing staff actively guide patients to adopt a positive attitude towards the disease, build confidence in overcoming it, and encourage active cooperation with rehabilitation treatment. Meanwhile, psychological counseling is provided to family members, accompanied by comprehensive health education on disease-related knowledge. This aims to alleviate family anxiety and help them establish reasonable expectations for subsequent treatment and rehabilitation.

Provide the child with a comfortable environment, including appropriate temperature, humidity, and soft lighting. Nursing staff should provide early guidance, help family members fully familiarize themselves with the next steps of functional exercises, and guide the child to perform these exercises. Active exercise of the affected limb can reduce swelling and effectively relieve pain. Medical staff should encourage parents to actively participate in the child's treatment and management, ensure that parents fully understand the child's condition, guide parents to understand and tolerate the abnormal behaviors of children caused by pain, and advise parents to use more words of encouragement and praise. Increased companionship is beneficial to alleviating pain.

### 2.4 Observational indicators

#### 2.4.1 Primary outcome

##### 2.4.1.1 NRS pain score (postoperative pain score at 6 h and 24 h)

The Numerical Rating Scale (NRS) was adopted for the assessment of postoperative pain. Pain assessment was performed by the primary nurses. NRS is a scale that measures pain intensity using 11 numerical values ranging from 0 to 10. Patients were instructed to select the number that best reflected their pain level, where 0 represents “no pain” and 10 represents “the most severe (unbearable) pain”.

#### 2.4.2 Secondary outcome

##### 2.4.2.1 Length of hospital stay and postoperative complications

Length of hospital stay was defined as the number of days from admission to discharge. Postoperative complications included postoperative Kirschner wire breakage, incision infection, malunion, fracture nonunion, and osteofascial compartment syndrome. Complications were assessed during hospitalization and at follow-up visits at 1, 3, and 6 months postoperatively.

##### 2.4.2.2 Self-rating anxiety scale (SAS)

The Self-rating Anxiety Scale (SAS) was used for assessment. This scale consists of 20 items, adopting a 4-point Likert scale, with each item scored from 1 to 4 points. The standard score is 1.25 times the sum of all item scores, rounded to the nearest integer, and the total score of the scale ranges from 25 to 100 points. With a cut-off score of 50, standard scores >50 indicate the presence of anxiety: 50–59 points for mild anxiety, 60–69 points for moderate anxiety, and ≥70 points for severe anxiety (13). This scale was administered to the accompanying caregivers by the primary nurse at the time of the child's enrollment (pre-intervention) and 24 h postoperatively (post-intervention).

##### 2.4.2.3 Tampa scale for kinesiophobia-11 (TSK-11)

The Tampa Scale for Kinesiophobia-11 (TSK-11) is a brief, reliable two-factor instrument, consisting of 11 items and employing a 4-point Likert scale (ranging from 1 = strongly disagree to 4 = strongly agree) (14). The items are designed to assess the fear of movement/re-injury induced by pain. Across a range of adult pain populations, the internal consistency of the two factors ranges from 0.64 to 0.80.

##### 2.4.2.4 Postoperative functional exercise compliance questionnaire

Postoperative functional exercise compliance of pediatric patients was assessed using the Postoperative Functional Exercise Compliance Questionnaire. The questionnaire encompasses six aspects: knowledge of postoperative functional rehabilitation, implementation of medical advice, duration of daily functional exercise, frequency of daily exercise, initiative in exercising, and ability to perform activities of daily living. A 5-point scoring system was applied to each item, with scores ranging from 0 to 4; a higher score indicates better compliance. Complete compliance was determined as achieving a score of 3 or above for all items; otherwise, the patient was classified as non-compliant (15, 16).

The Self-rating Anxiety Scale (SAS), Tampa Scale for Kinesiophobia-11 (TSK-11), and Postoperative Functional Exercise Compliance Questionnaire were collected by a nurse who was not involved in the intervention and was blinded to group allocation.

### 2.5 Statistical methods

Statistical analysis of the data was performed using SPSS 22.0 software. Quantitative data in the results were expressed as mean ± standard deviation or median (IQR), as appropriate. For normally distributed quantitative data, intergroup comparisons were conducted using an independent samples t-test with mean difference (MD) and 95% confidence interval (CI) reported; for non-normally distributed quantitative data, intergroup comparisons were performed using the Mann–Whitney U test with median difference (Hodges–Lehmann estimate) and 95% CI reported. ANCOVA was applied to compare postoperative SAS scores, with preoperative SAS score as the covariate. Categorical data were presented as percentages (%), and intergroup comparisons were carried out using the Chi-square test (*χ*^2^ test), or Fisher's exact test when expected frequencies were <5, with risk ratio (RR) and 95% CI reported for compliance outcomes. A *P*-value < 0.05 was considered statistically significant.

## 3 Results

### 3.1 Clinical characteristics and baseline comparison

A total of 120 pediatric patients with limb fractures were enrolled and divided into two groups: 60 patients admitted from January 1 to August 20, 2024 were assigned to the control group, and 60 patients admitted from August 21, 2024 to May 31, 2025 were assigned to the intervention group.

There were no statistically significant differences in the baseline data between the two groups of children (*P* > 0.05) (Table 2). The difference in the distribution of detailed fracture sites between the two groups was not statistically significant (Fisher‘s exact test, *P* = 0.378). The detailed fracture sites are presented (Table 3). There were 2 cases of open fractures in the control group and none in the intervention group(Fisher‘s exact test, *P* = 0.496). No postoperative complications, including Kirschner wire breakage, incision infection, malunion, nonunion of fracture, or osteofascial compartment syndrome, were observed in either group. Children in both groups received at least one 100-mg ibuprofen suppository within the first 6 postoperative hours. No other postoperative analgesics were administered. Medication data between 6 and 24 h postoperatively were incomplete; therefore, these data were not included in the analysis.

**Table 2:** Comparison of the baseline data between the two groups of children.

Variable	Gender		Age	Fracture Site		Main Caregiver			Main Caregiver Educational Level		
	Male	Female		Upper Limb	Lower Limb	Parents	Grandparents	Other Family Members	High School or Below	Junior College	Bachelor's Degree or Above
Control Group	43 (71.7%)	17 (28.3%)	9 (8,11.75)	37 (61.7%)	23 (38.3%)	51 (85.0%)	7 (11.7%)	2 (3.3%)	6 (10.0%)	25 (41.7%)	29 (48.3%)
Intervention Group	42 (70.0%)	18 (30.0%)	10 (8,12)	39 (65.0%)	21 (35.0%)	49 (81.7%)	7 (11.7%)	4 (6.7%)	5 (8.3%)	21 (35.0%)	34 (56.7%)
*χ*^2^/U	0.04		2,002	0.144		0.707			0.836		
P	0.841^a^		0.284^b^	0.705^a^		0.702^a^			0.659^a^		

a the Chi-square test (χ^2^ test).

b the Mann–Whitney U test.

**Table 3:** Comparison of fracture site distribution between the two groups.

Fracture Sites	Control Group	Intervention Group
Radius	12	9
Ulna	3	6
Radius and Ulna	5	5
Finger	1	5
Humerus	16	14
Tibia	6	12
Femur	9	7
Fibula	1	1
Tibia and Fibula	3	0
Ankle	2	1
Patella	1	0
Calcaneus	1	0
Total	60	60

### 3.2 Primary outcomes

The primary outcomes are summarized (Table 4), and the secondary outcomes are presented (Table 5).

**Table 4:** Comparison of primary outcome indicators (pain scores at 6 h and 24 h postoperatively) between the two groups.

Variable	pain scores at 6h	pain scores at 24h
Control Group	5 (4,5)	3 (2,4)
Intervention Group	4 (3,5)	1 (0,2)
U	1,073.5	402
Median difference (95% CI)	0.99994 (0.00005–1.00000)	2.00 (2.00–2.00)
P	<0.001	<0.001

**Table 5:** Comparison of secondary outcome indicators between the control and intervention groups.

Variable	hospital stay(day)	TSK scores	compliance rate with functional exercises	Preoperative SAS score	Postoperative SAS score
Control Group	4 (2,5)	37.10 ± 2.71	58.3%	70 (70, 70.75)	63.5 (63,63.75)
Intervention Group	3 (2,3),	24.65 ± 2.43	86.7%	70 (69,70)	42 (40,44),
T/χ^2^/U	1,224	26.505	12.079	1,644	12
P	0.002^b^	<0.001^c^	0.001^a^	0.377^b^	<0.001^b^

a the Chi-square test (χ^2^ test).

b the Mann–Whitney U test.

c independent samples t-test.

The median pain score at 6 h postoperatively in the intervention group was 4 (IQR: 3, 5), compared with 5(IQR: 4,5) in the control group (Figure 1). The difference was statistically significant (U = 1,073.5, Z = −4.025, *P* < 0.001), with an effect size of r = 0.367, Hodges–Lehmann median difference = 0.99994 (95% CI: 0.00005–1.00000; control minus intervention).

**Figure 1:**
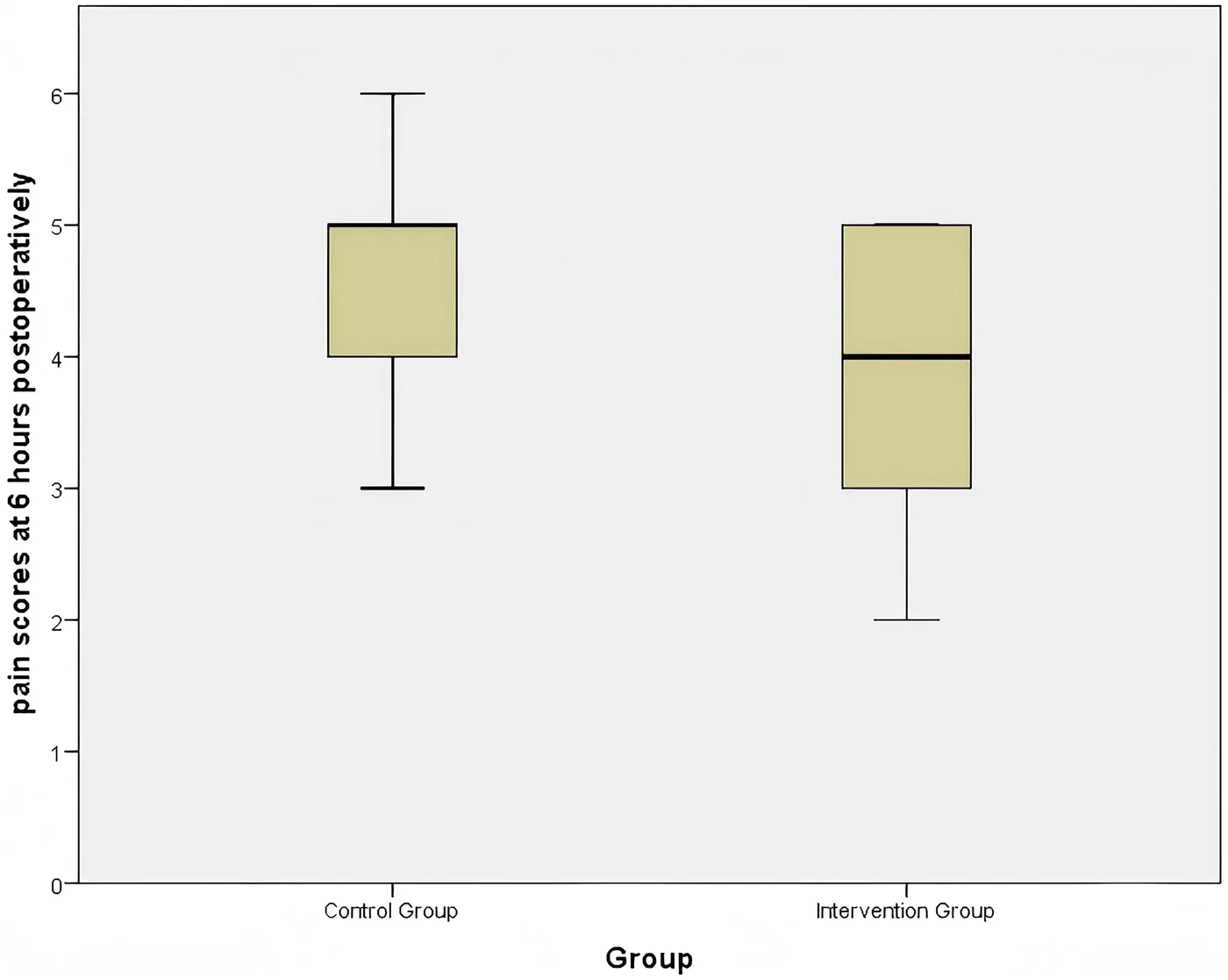
Box plot of 6-hour postoperative pain scores in the control and intervention groups.

The median pain score at 24 h postoperatively in the intervention group was 1 (IQR: 0, 2), compared with 3 (IQR: 2,4) in the control group (Figure 2). The difference was statistically significant (U = 402, Z = −7.519, *P* < 0.001), with an effect size of r = 0.685, Hodges–Lehmann median difference = 2.00 (95% CI: 2.00–2.00; control minus intervention).

**Figure 2:**
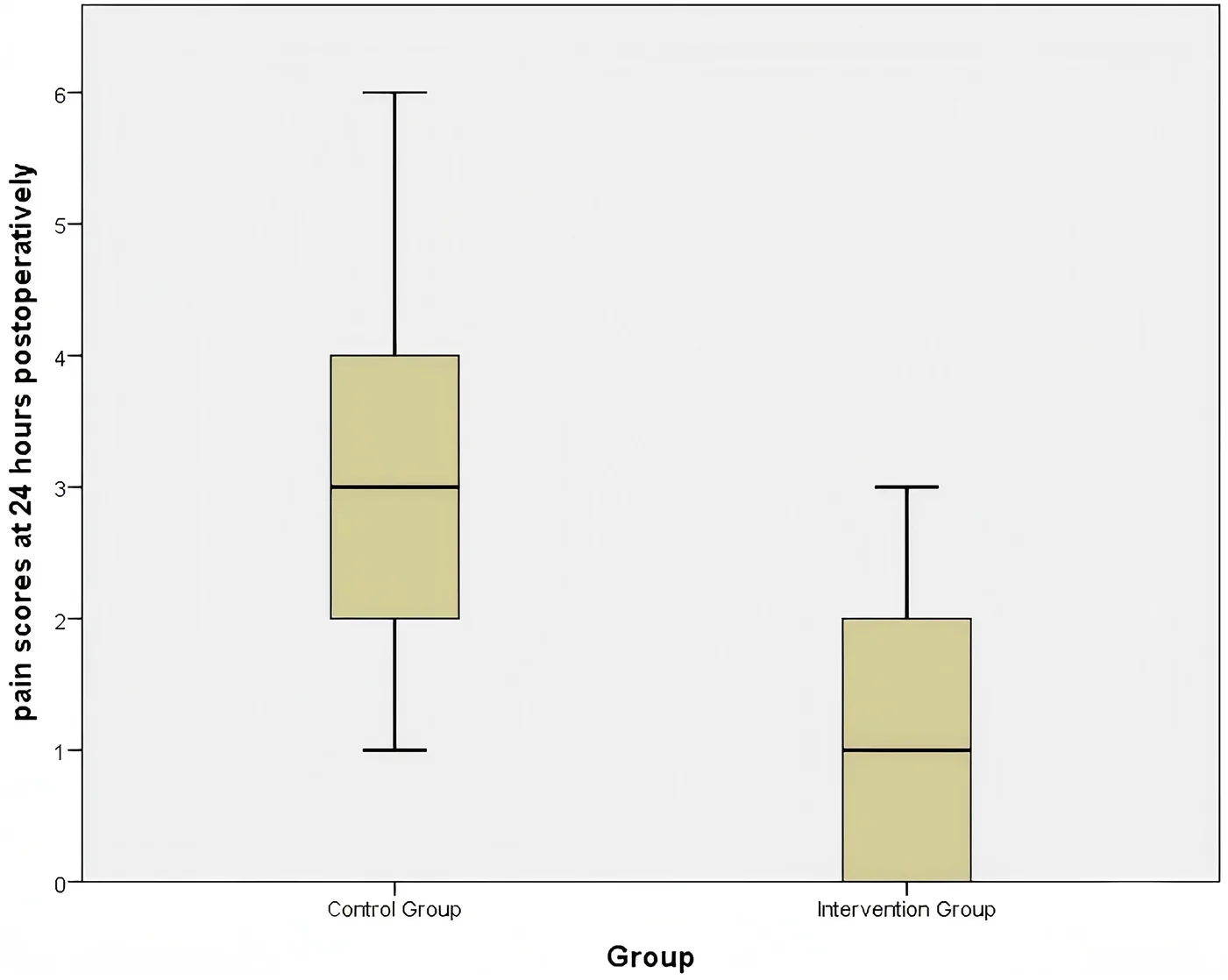
Box plot of 24-hour postoperative pain scores in the control and intervention groups.

### 3.3 Secondary outcome

The median length of hospital stay in the intervention group was 3 (IQR: 2,3), compared with 4 (IQR: 2,5) in the control group. The difference was statistically significant (U = 1,224, Z = −3.099, *P* = 0.002), with an effect size of r = 0.283.

The TSK scores in the Intervention Group (24.65 ± 2.43) were significantly lower than those in the Control Group (37.10 ± 2.71), with statistically significant differences (MD = 12.45，95% CI：11.520–13.380, t = 26.505, *P* < 0.001).

The intervention group had a significantly higher compliance rate of functional exercises than the control group (86.7% vs. 58.3%; *χ*^2^ = 12.079, *P* = 0.001). The RR for complete compliance was 0.673 (95% CI: 0.532–0.852),for the control group relative to the intervention group.

The median preoperative SAS score in the intervention group was 70 (IQR: 69,70), compared with 70 (IQR: 70,70.75) in the control group. The difference was not statistically significant (U = 1,644, Z = −0.883, *P* = 0.377), with an effect size of r = 0.081.

The median postoperative SAS score in the intervention group was 42 (IQR: 40,44), compared with 63.5 (IQR: 63,63.75) in the control group. The difference was statistically significant (U = 12, Z = −9.416, *P* < 0.001), with a Hodges—Lehmann median difference of 22.00 (95% CI: 20.00 to 23.00; control minus intervention) and an effect size of r = 0.860. The group × preoperative SAS score interaction was not significant (F = 0.401, df = 1, *P* = 0.528), so the final ANCOVA model included main effects only. After adjustment for preoperative SAS score, the adjusted mean difference was 20.87 (95% CI: 19.38 to 22.37,control minus intervention, F = 764.58, df = 1, *P* < 0.001). Both analyses were consistent in direction and magnitude.

## 4 Discussion

At present, pain has been widely recognized as the fifth vital sign, in addition to body temperature, pulse, respiration, and blood pressure. Severe pain may lead to a series of severe complications, and adversely affect therapeutic efficacy and prognosis. Accordingly, pain management in orthopedic patients has gained increasing clinical attention, and appropriate analgesic treatment has been increasingly regarded as a human right (7, 17). However, the routine nursing model lacks evidence-based and individualized interventions, which can neither satisfy patients’ growing care demands nor yield expected clinical effects. Therefore, we developed a nurse-led and family-involved pain management program for children with limb fractures.

Clinical pain management in China is currently primarily delivered under a nurse-led model (6). As frontline clinical providers, nurses engage in more frequent communication with pediatric patients and their families than physicians do. Existing literature has demonstrated the efficacy of nurse-led nonpharmacological interventions in pain management (10). Nurse-led counseling and postoperative care can effectively relieve pain and improve patient satisfaction (18).

In pain management, accurate pain assessment constitutes the first step, as it provides essential information for pain control and helps evaluate the efficacy of analgesic treatment (7). Kang et al. (19) reported that parental pain assessment demonstrates favorable reliability in evaluating pain in pediatric patients. However, family members tend to overestimate children's pain, whereas medical staff are prone to underestimating it. Therefore, the role of family assessment should not be overlooked to achieve more accurate pain evaluation. Furthermore, given the young age of pediatric patients, family members provide essential support for functional exercise, as well as emotional and familial care, all of which play a vital role in children's rehabilitation. Therefore, the involvement of family members in pain management for children should not be underestimated. In addition, a study by Wang et al. (20) confirmed the efficacy of preoperative education in reducing postoperative pain. The results of the present study demonstrate that a nurse-led, family-involved pain management program was associated with reduced postoperative pain in pediatric patients. The underlying mechanisms are as follows: ① This program establishes a dedicated pain management team and trains nurses in pain management principles and evidence-based analgesic approaches. ② While medical staff generally deliver one-to-many care without constant bedside presence, family members provide multi-to-one care to pediatric patients and remain by their bedside most of the time. This enables family members to identify pain-related abnormalities earlier and more promptly than medical staff. ③ Following enhanced preoperative education, family members can implement accurate and timely pain relief measures and seek medical intervention when appropriate.

Pain itself can elevate fear levels in pediatric patients, and children undergoing surgery typically experience even higher levels of anxiety. Without effective management, such fear may lead to poor treatment compliance in children, aversion to postoperative rehabilitation exercises and reluctance to perform normal activities, ultimately resulting in delayed return to daily activities, and prolonged hospital stays (21). In this study, the main caregivers of the pediatric patients were their parents, who are often the primary earners of the family. The financial burden resulting from the illness, the disruption of the family's normal daily routine, as well as the children's restlessness, crying, and noncompliance with treatment caused by pain, all contribute to heightened anxiety among family caregivers. The findings of this study suggest that the nurse-led, family-involved pain management program was associated with better pain control, reduced fear of movement, and improved adherence to rehabilitation exercises. These changes were accompanied by shorter hospital stays, indicating a potential benefit of this model in facilitating postoperative recovery. Meanwhile, comprehensive preoperative education equips family members with relevant medical knowledge, enhances their communication with medical staff, and fosters reasonable expectations toward treatment. This facilitates their active involvement in care, improves the transparency of treatment-related information, effectively relieves anxiety among family caregivers, and further increases their satisfaction with nursing services.

This study has several limitations. First, the quasi-experimental design with a relatively small sample size and non-concurrent enrollment limits causal inference, as the two groups were recruited during different calendar periods, making it difficult to separate the intervention effect from temporal or seasonal trends. However, the two groups were comparable in age, sex, and fracture type (all *P* > 0.05), which does not rule out the possibility that time-related factors drove the observed differences. Moreover, we cannot rule out unmeasured shifts in care practices over the study period. Second, despite the use of standardized assessment protocols, the lack of blinding (the primary nurses were aware of group allocation) may have introduced detection bias. Third, neither the TSK-11 nor the compliance questionnaire has been formally validated in Chinese children with fractures; therefore, the results obtained from these instruments should be interpreted as exploratory. Fourth, multiple comparisons were performed without adjustment for multiplicity, so secondary outcomes should be interpreted with caution. Finally, the single-center design limits the generalizability of our findings, and the inability to compare the exact timing and cumulative doses of ibuprofen between groups is an additional limitation of this study. These limitations should be considered when interpreting the results, and future multicenter randomized controlled trials with blinded outcome assessment are warranted to confirm the effectiveness of this program.

In summary, The nurse-led, family-involved pain management program was associated with clinically meaningful benefits across multiple outcomes. Compared with routine nursing care, this model demonstrated advantages in reducing postoperative pain, alleviating fear of movement, improving exercise compliance, and decreasing family anxiety. These findings suggest that integrating nurse-led and family-involved approaches into postoperative care may represent a practical and potentially beneficial approach for children with limb fractures.

## Data Availability

The raw data supporting the conclusions of this article are not publicly available due to patient privacy and ethical restrictions. The data will be made available by the corresponding author upon reasonable request. Requests to access the datasets should be directed to Jinrun Lin, 339685510@qq.com.
